# Low utility of blood culture in pediatric community-acquired pneumonia: An observational study on 2705 patients admitted to the emergency department

**DOI:** 10.1097/MD.0000000000007028

**Published:** 2017-06-02

**Authors:** Jae Hyun Kwon, Jung Heon Kim, Jeong-Yong Lee, Youn-Jung Kim, Chang Hwan Sohn, Kyoung Soo Lim, Won Young Kim

**Affiliations:** a Department of Pediatrics, University of Ulsan College of Medicine, Asan Medical Center; b Department of Emergency Medicine, University of Ulsan College of Medicine, Asan Medical Center, Seoul, Korea.

**Keywords:** bacteremia, blood culture, pediatric emergency medicine, pneumococcal infections, pneumonia

## Abstract

To investigate the utility of blood cultures performed on previously healthy children and adolescents with community-acquired pneumonia (CAP) at a tertiary care hospital emergency department (ED).

We reviewed 3235 patients with CAP aged 6 months to 18 years who underwent blood cultures at the ED from 2009 through 2016. CAP was defined according to the International Classification of Diseases, 10th Revision codes for pneumonia and the requirement of antibiotic treatment plus any of the following: radiologically confirmed, hospitalized, or moderate to severe disease. Blood cultures were retrospectively justified by the Infectious Diseases Society of America guidelines. We measured the yield (true positive results of blood culture) and impact (blood culture-directed change in the antibiotic regimen).

Of 2705 previously healthy patients with CAP, 833 (30.8%; 95% confidence interval [CI]: 29.1–32.6) underwent blood cultures justified by the current guidelines. We found 12 patients (0.4%; 95% CI: 0.2–0.8) having positive results of blood culture, 7 of whom underwent justified blood cultures. Of these 7 patients, 3 (0.11%; 95% CI: 0.02–0.3) had the yield, *Streptococcus pneumoniae*. No impact was made in these 3 patients with *S pneumoniae*.

We confirmed a low utility of blood cultures in previously healthy children and adolescents with CAP who were admitted to the ED. This finding suggests the need to refine the current guidelines for obtaining blood cultures in the ED for pediatric CAP.

## Abstract 2

Supplemental Digital Content is available in the text

## 1 Introduction

The Infectious Diseases Society of America recommends that blood culture (BC) should be limited to children and adolescents (hereafter designated as children, unless otherwise specified) with moderate to severe, presumed bacterial community-acquired pneumonia (CAP).^[1]^ The recent studies performed on hospitalized children with CAP show a prevalence of bacteremia, ranging from 1.1% to 7.0%,^[2–5]^ and a BC-directed change in the antibiotic regimen of 4.6%.^[4]^ However, the Infectious Diseases Society of America recommendation is based on low-quality evidence, and BC is still frequently performed on hospitalized children with CAP.^[5,6]^ The overuse of BC can lead to unnecessary venipuncture and antibiotic treatment.

In the emergency department (ED), BC is still frequently performed as an initial workup for CAP.^[7,8]^ A recent study performed on 238 children with CAP who underwent a BC at the ED shows 9 cases of bacteremia without any BC-directed change in the antibiotic regimen.^[9]^ Despite this, there is a lack of ED-based studies on the utility of BC in pediatric CAP with larger study populations.

In the ED, BC may not need to be performed on all children with moderate to severe CAP. We aimed to investigate the utility of BC in children with CAP who were admitted to the ED of a tertiary care hospital. We also compared the utility of BC in children with and without a BC justified by the current guidelines.

## 2 Methods

### 2.1 Study design and setting

This retrospective study was conducted at a tertiary care, university-affiliated hospital ED in Seoul, Korea, which has an annual census of approximately 40,000 children. We reviewed previously healthy children with CAP aged 6 months to 18 years as a primary diagnosis who were admitted to the ED and underwent a BC for the diagnosis of bacteremia from January 2009 through September 2016. At our institution, children with symptoms suggestive of CAP frequently undergo BC as part of an initial workup. During an epidemic, children undergo testing for viruses or *Mycoplasma pneumoniae*. Children aged <6 months were excluded since they were partially immunized with pneumococcal conjugate vaccine (PCV) and were likely to benefit from BC given their susceptibility to bacteremia. In Korea, 10- and 13-valent PCV (PCV10/13) were licensed in 2010, and were included in the national immunization program in 2014. It is speculated that at least 65% and 40% of the children underwent the immunization and recent antibiotic treatment, respectively.^[10,11]^ The study protocol was approved by the institutional review board with a waiver for informed consent (IRB No. 2016-1204).

### 2.2 Definitions and exclusion criteria

To investigate the utility of BC, we measured the yield and impact. The yield was defined as the presence of a single pathogenic bacterium from the blood, excluding contaminants. The impact was defined as a BC-directed change in the antibiotic regimen. CAP was defined according to the International Statistical Classification of Diseases and Related Health Problems, 10th Revision codes for pneumonia (J10–J18) and the requirement of antibiotic treatment, implying the presumed bacterial CAP, plus any of the following: consolidation, peribronchial infiltration or pleural effusion on a chest radiograph; hospitalization; or moderate to severe disease according to the current guidelines.^[1]^

We excluded the children for any of the following: known cardiopulmonary diseases; presumed viral or noninfectious pneumonia; nonambulatory status (e.g., cerebral palsy); known immunocompromised status; indwelling devices (e.g., tracheostomy tube); and healthcare-associated pneumonia, which was confirmed >48 h after ED presentation or <2 weeks after discharge from a hospital.

### 2.3 Performance, justification, and interpretation of BC

BC was performed using BACTEC FX (Becton, Dickinson and Company, Franklin Lakes, NJ). During the study period, it was a routine procedure that an attending physician or nurse aseptically draws a sample of blood, and inoculates the sample into a pair of aerobic and anaerobic bottles (≥1 mL for each bottle). BC was retrospectively justified if it had been performed on children with respiratory distress (as defined in Section 2.5), admission to the intensive care unit or a complicated pneumonia.^[1]^ Pathogenic bacteria were defined as one of the following: *Streptococcus pneumoniae*, *Staphylococcus aureus*, group A beta-hemolytic streptococci, and *Haemophilus influenzae*. Contaminants were defined as one of the following: coagulase-negative staphylococci, viridans group streptococci, *Bacillus* spp., *Micrococcus* spp., *Propionibacterium* spp., and *Corynebacterium* spp. If no growth was reported after 5 days of BC, it was considered negative.

### 2.4 Testing for viruses or *M pneumoniae*

Viral or atypical etiology should be mentioned in pediatric CAP since these etiologies are more common than bacteria. We used a virus real-time polymerase chain reaction using CFX96 (BIORAD, Hercules, CA) and an influenza antigen test using Sofia Fluorescent Immunoassay Analyzer (Quidel, San Diego, CA) from the nasopharyngeal secretion. A *M pneumoniae* IgM chemiluminescence immunoassay was performed using LIAISON *M pneumoniae* IgM (DiaSorin S.p.A., Saluggia, Italy) from the blood.

### 2.5 Data collection

We used standardized data collection sheets. Clinical characteristics including age, gender, body temperature, signs of respiratory distress (age-adjusted tachypnea, chest retraction, and oxyhemoglobin saturation <90%), and outcome (admission to general wards or intensive care units, transfer, and death) were obtained. Age-adjusted tachypnea was defined as a respiratory rate >50/min in children aged 6 to 12 months, >40/min in those aged 1 to 5 years, and >20/min in those aged >5 years.^[1]^ Concentrations of inflammatory biomarkers including white blood cell, absolute neutrophil count, and C-reactive protein were recorded. For children with multiple visits, information on the earliest visit was used.

### 2.6 Statistical analysis

For comparison of clinical characteristics between the children with and without justified BC, Student *t* tests or Mann–Whitney *U* tests were used for continuous variables, and chi-squared tests or Fisher exact tests for categorical variables. A *P* of <.05 was considered statistically significant. Statistical analysis was performed using SPSS for Windows version 21.0 (SPSS Inc, Chicago, IL).

## 3 Results

### 3.1 Study population

**Figure 1:**
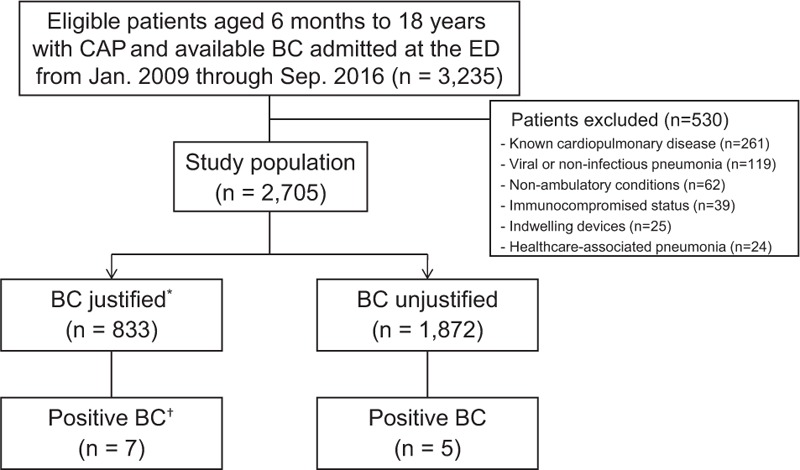
Flowchart for the selection of patients. ^∗^Assessed by the Infectious Diseases Society of America guidelines.^[1]^^†^The 3 children with the yield belong here. BC = blood culture, CAP = community-acquired pneumonia, ED = emergency department.

From a total of 3235 children with CAP who underwent BC at the ED during the study period, 2705 met all inclusion criteria (Fig. 1). Table 1 outlines the clinical characteristics. Overall, BC was justified in 833 children (justified BC group: 30.8%; 95% confidence interval [CI]: 29.1–32.6). These children had older age and higher frequency of male gender. They also had higher frequencies of admission and *M pneumoniae*, and higher concentration of C-reactive protein. BC was justified mainly by the respiratory distress (89.4%). Among the children with respiratory distress (n = 745), we found that 597 had age-adjusted tachypnea, 230 had chest retraction, and 20 had oxyhemoglobin saturation <90% (mutually inclusive). Thirty-five admissions to the intensive care unit and 4 deaths were noted.

**Table 1:**
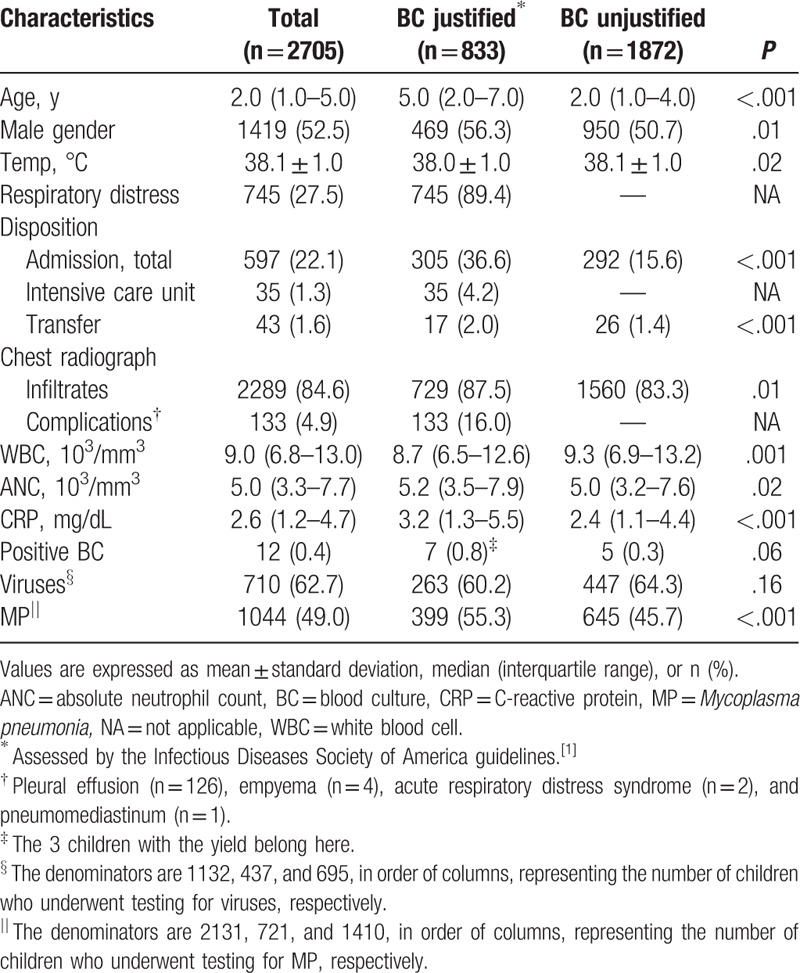
Clinical characteristics of the study population.

### 3.2 Yield and impact

Of 2705 children, 12 (0.4%; 95% CI: 0.2–0.8) had positive results of BC (Table 2). Of these, only 3 children (0.11%; 95% CI: 0.02–0.3) had the yield, *S pneumoniae*, and 2 of whom had this bacterium from BCs performed at referring hospitals without additional yield following referral to the ED. No impact was found in these 3 children. The other 9 children were considered to have contaminants (overall contamination rate: 0.3%; 95% CI: 0.2–0.6). Hence, in our cohort, the prevalence of bacteremia was 0.11% without any BC-directed change in the antibiotic regimen.

**Table 2:**
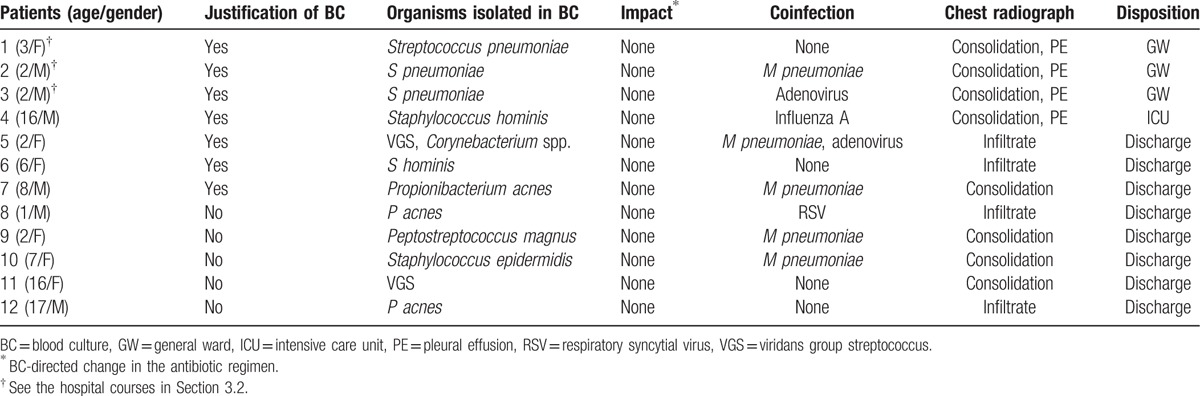
Characteristics of the patients with positive results of blood culture.

The girl aged 3 years with the yield (patient 1, Table 2) had CAP manifested as consolidation and pleural effusion. Subsequently, she underwent antibiotic treatment and drainage of the pleural effusion, which was proven sterile on culture. After 27 days of admission in the general ward, she was discharged without complications. Two boys with the yield from referring hospitals (patients 2 and 3, Table 2) had similar hospital courses, except that patient 3 had atelectasis as a sequela. All 3 children had CAP from 2010 through 2012 before PCV10/13 were included in the Korean national immunization program.

Of 2 children who had positive results of BC among the 530 excluded children, 1 with known ventricular septal defect had a yield, methicillin-resistant *S aureus*, without an impact. This child had been presumed as having transient bacteremia, and recovered without antibiotic treatment.

### 3.3 Viruses and *M pneumoniae*

One thousand one hundred thirty-two (41.8%) and 2131 (78.8%) children, primarily hospitalized, underwent testing for viruses and *M pneumoniae*, respectively (Table 1). We were unable to have a complete dataset with these etiologies. *M pneumoniae* was the single, most common pathogen in this study (38.6%, 1044 of the 2705 children). Among the viruses detected, rhinovirus was most common, followed by respiratory syncytial virus (see Table, Supplemental Digital Content, which shows the viral etiology). Overall, 64.8% (1467 of the 2263 children who underwent testing for viruses or *M pneumoniae*) had a coinfection of viruses or *M pneumoniae*, and this coinfection was more frequent in the justified BC group (70.9%, 537 of the 757 children vs. 61.8%, 930 of the 1506 children, *P* < .001).

## 4 Discussion

We found a low utility of BC for the diagnosis of bacteremia in a highly selected population, consisting of previously healthy children aged 6 months to 18 years with CAP who were admitted to the ED of a tertiary care hospital. To the best of our knowledge, this study used the largest ED-based study population for studies on this topic.^[7,9]^ The prevalence of bacteremia (0.11%) indicates that the number needed to test was approximately 909. Judicious use of BC can decrease unnecessary venipuncture and antibiotic treatment.^[1,12]^

Our yield was lower than the prevalence of bacteremic CAP in the post-PCV10/13 era, ranging from 1.1% to 1.5%.^[5,6]^ This discrepancy was likely due to the exclusion of underlying medical conditions, along with the lower probability of selection bias in our cohort. The age of the children with bacteremia (2–3 years) corroborates the previous reports.^[7,13]^ The median age and gender predominance were comparable to a population-based study in the United States.^[14]^ Older age and male predominance in the justified BC group (i.e., moderate to severe disease) are parallel to the characteristics of critically ill children.^[15,16]^ The older age and higher frequency of *M pneumoniae* in this group can be partially explained, as this bacterium is more prevalent in children aged >5 years.^[14]^ The younger age in the unjustified BC group agrees with a study showing that younger children with CAP undergo BC more frequently.^[17]^

The low yield suggests the need to refine the current guidelines for obtaining BC in the ED, with an emphasis on the risk factors of bacteremia rather than disease severity itself. The justified BC group still had low yield, suggesting that moderate to severe disease does not necessarily mean the potential of bacteremia. The justified BC group showed a higher frequency of coinfection of viruses or *M pneumoniae*. Findings of the inflammatory biomarkers and body temperature oppose higher bacteremic risk in the justified BC group. Although the concentration of C-reactive protein in this group was higher, the difference of median values was small, and the white blood cell count and body temperature showed opposite patterns. The increasing use of PCV10/13 is decreasing the prevalence of invasive pneumococcal disease,^[18]^ which may further decrease the utility of BC. This change can be exemplified by the characteristics of the 3 children with bacteremia who visited the ED in the pre-PCV10/13 era in Korea. The increased incidence of *M pneumoniae* in younger children indicates a further decrease in the utility of BC.^[19,20]^

Several limitations related to the single center, retrospective design are worth mentioning. It is unknown whether there was a high suspicion of bacterial CAP at presentation. Some cases of viral CAP or bronchiolitis might be logically excluded by the definition of CAP used in this study. Still, the proportion of children having viruses (26.2%) implies some degree of misclassification. Although BC was justified according to the current guidelines,^[1]^ some criteria such as the Pediatric Early Warning Score were unable to be assessed. The single-centered setting possibly mitigates the strength of the largest ED-based study population. However, this matter might be less important given the probably low selection bias compared to previous studies.

In summary, we found a low utility of BC in previously healthy children with CAP at the ED. This study encourages the refinement of the current guidelines for obtaining BC from children with CAP in the ED. Future studies may focus on determining the risk factors of bacteremia in previously healthy children with CAP and the utility of polymerase chain reaction for the diagnosis of *S pneumoniae* bacteremia.

## Acknowledgment

The authors appreciate the statistical consultation of Minju Kim, a statistician.

## Supplementary Material

Supplemental Digital Content
