# Supplementary material for: Low utility of blood culture in pediatric community-acquired pneumonia: An observational study on 2705 patients admitted to the emergency department
Source: Medicine (Baltimore). 2017 Jun 2;96(22):e7028. doi: 10.1097/MD.0000000000007028 (PMC5459721; doi:10.1097/MD.0000000000007028)
Supplement: Supplemental Digital Content [file medi-96-e7028-s001.docx]

**Supplemental Digital Content**Viral etiology (*n* = 710)

| **Viruses** | **Total**  **(*n* = 710)** | **BC justified**^*^  **(*n* = 263)** | **BC unjustified**  **(*n* = 447)** | ***P*** |
| --- | --- | --- | --- | --- |
| Rhinovirus | 226 (31.8) | 82 (31.2) | 144 (32.2) | .78 |
| RSV | 206 (29.0) | 68 (25.9) | 138 (30.9) | .16 |
| Adenovirus | 132 (18.6) | 49 (18.6) | 83 (18.6) | .98 |
| Parainfluenza | 104 (14.6) | 34 (12.9) | 70 (15.7) | .32 |
| HMPV | 90 (12.7) | 24 (9.1) | 66 (14.8) | .03 |
| Influenza | 77 (10.8) | 44 (16.7) | 33 (7.4) | < .001 |
| Others^†^ | 134 (18.9) | 48 (18.3) | 86 (19.2) | .75 |
| Coinfection | 409 (57.6) | 157 (59.7) | 252 (56.4) | .39 |
| With viruses**^‡^** | 216 (30.4) | 71 (27.0) | 145 (32.4) | .13 |
| With bacteria^§^ | 289 (40.7) | 125 (47.5) | 164 (36.7) | .01 |
